# The non-invasive documentation of coronary microcirculation impairment: role of transthoracic echocardiography

**DOI:** 10.1186/1476-7120-3-18

**Published:** 2005-08-04

**Authors:** Pawel Petkow Dimitrow, Maurizio Galderisi, Fausto Rigo

**Affiliations:** 1 2^nd ^Department of Cardiology, Collegium Medicum, Jagiellonian University, Cracow, Poland; 2 Division of Cardioangiology with CCU, Department of Clinical and Experimental Medicine, Federico II University of Naples, Italy; 3 Department of Cardiology Umberto I° Hospital Mestre-Venice, Italy

## Abstract

Transthoracic Doppler echocardiographic-derived coronary flow reserve is an useful hemodynamic index to assess dysfunction of coronary microcirculation. Isolated coronary microvascular abnormalities are overt by reduced coronary flow reserve despite normal epicardial coronary arteries. These abnormalities may occur in several diseases (arterial hypertension, diabetes mellitus, hypercholesterolemia, syndrome X, aortic valve disease, hypertrophic cardiomyopathy and idiopathic dilated cardiomyopathy). The prognostic role of impaired microvascular coronary flow reserve has been shown unfavourable especially in hypertrophic or idiopathic dilated cardiomyopathies. Coronary flow reserve reduction may be reversible, for instance after regression of left ventricular hypertrophy subsequent to valve replacement in patients with aortic stenosis, after anti-hypertensive treatment or using cholesterol lowering drugs. Coronary flow reserve may increase by 30% or more after pharmacological therapy and achieve normal level >3.0. In contrast to other non invasive tools as positron emission tomography, very expensive and associated with radiation exposure, transthoracic Doppler-derived coronary flow reserve is equally non invasive but cheaper, very accessible and prone to a reliable exploration of coronary microvascular territories, otherwise not detectable by invasive coronary angiography, able to visualize only large epicardial arteries.

## The concept of coronary flow reserve (CFR) and transmural reduction of CFR

**Figure 1:**
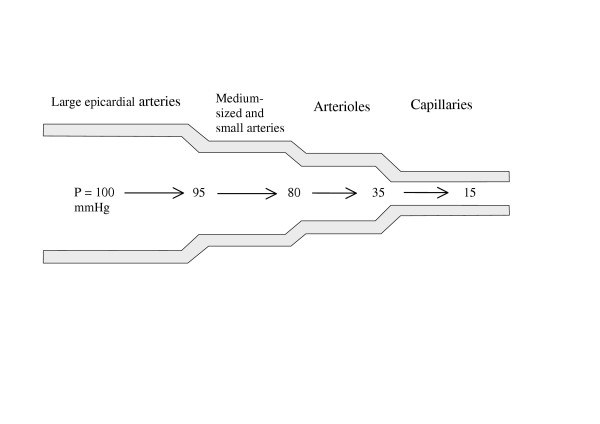
Sequential decrease of coronary perfusion pressure in consecutive segments of coronary vasculature. The largest fall in perfusion pressure occurs in coronary resistance arterioles.

The coronary arterial tree consists of four basic segments. Epicardial coronary arteries give off small transmural penetrating arteries, which have branches in the myocardial layers. These branches are defined arterioles, terminating in capillary vessels, directly supplying myocardial cells. Each of these coronary segments produces different level and degree of resistance to coronary blood flow. Normal (non-stenosed) large epicardial coronary arteries play a minor role in the regulation of coronary vascular resistance and act mainly as conductance (conduit) vessels. Most of the resistance, which opposes coronary blood flow, arises from resistance arterioles. The resistance is manifest by decreased coronary perfusion pressure. The percent distribution of the length and resistance of individual segments of the coronary vascular tree is summarised in Table 1 and depicted in Figure 1.

**Table 1:** Distribution of coronary resistance in normal coronary arterial bed.

	Large epicardial arteries	Medium-sized and small arteries		Arterioles	Capillaries
Diameter	>1000 μm	1000–100 μm.		100–10 μm	<10 μm
% of total resistance	5%		15–25% 400–100 μm	50–60%	20%
% length of coronary bed	5–10%	15–25%		60–75%	

**Figure 2:**
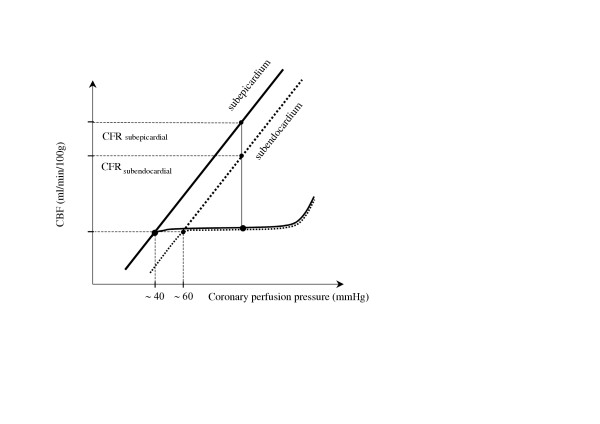
Transmural distribution of coronary flow reserve (CFR); CFR subepicardial > CFR subendocardial.

The difference between coronary blood flow corresponding to flow autoregulation plateau at rest and coronary blood flow after maximal vasodilatation is traditionally defined as coronary flow reserve (CFR) [1-6]. CFR is usually calculated as the ratio of maximal (hyperemic) to resting coronary blood flow (Figure 2). CFR is an important functional parameter to understand the pathophysiology of coronary circulation and can be used to examine the integrity of microvascular circulation.

The assessment of regional coronary flow reveals marked spatial heterogeneity of CFR across the myocardial wall. According to the model of Hoffman [2], the highest CFR is that measurable in the subepicardial layer of myocardium. In relation to a transmural flow reduction, CFR is significantly lower in the subendocardial layer, also due to elevated left ventricular (LV) diastolic pressure increasing extravascular compressive forces. As a result of this transmural coronary flow distribution, CFR is exhausted firstly in the subendocardial layer [2] (Figure 2). The lower limit of coronary flow autoregulation is unfavourably shifted to the higher value of coronary perfusion pressure in the subendocardial layer as compared with the subepicardial layer (i.e. 55–65 mm Hg versus 30–40 mmHg, respectively) [5] (Figure 2). According to Hoffman [2] the reduction of global CFR from 4.0 to 2.0 could be associated with the loss of flow reserve in a part or all of the subendocardial layer of the myocardium.

In human studies, due to limited spatial resolution of positron emission tomography (PET), the demonstration of subendocardial hypoperfusion has been made possible only in the hypertrophied myocardium [7]. In patients with aortic valve stenosis and normal epicardial coronary arteries [7], subendocardial CFR (1.43 ± 0.33) was lower than subepicardial CFR (1.78 ± 0.35; *P *= 0.01). In the subgroup of severe aortic stenosis (aortic valve area <0.8 cm^2^) CFR was <2.0 both at subendocardial and subepicardial levels and in two of these patients subendocardial CFR was <1.0 (lack of CFR).

An alternative approach to document transmural steal phenomenon is the calculation of subendocardial/subepicardial flow ratio [8,9]. A hyperemic value of subendocardial / subepicardial flow ratio <0.8 (i.e., subendocardial flow is at least 20% lower than subepicardial) has been proposed as marker of subendocardial hypoperfusion. In some patients with hypertrophic cardiomyopathy (HCM), subendocardial flow was 40% lower than subepicardial flow (ratio = 0.6) after infusion of a vasodilator agent [8,9].

Magnetic resonance imaging (MRI) with gadolinium contrast agent, has higher resolution and appears more sensitive providing the opportunity to assess even patients without left ventricular hypertrophy, i.e. with normal wall thickness [10]. Accordingly, cardiac MRI demonstrates subendocardial hypoperfusion in patients with syndrome X during the intravenous administration of adenosine when compared with healthy control subjects who showed increase of subendocardial perfusion after induced hyperemia [10].

## The lower limit of normal CFR and criteria for normal reference values

An important practical issue is to confirm in clinical studies that the lower limit of normal CFR is >3.0. This aspect is summarised in Table 2.

**Table 2:** Comparison of CFR using different methods in reference control groups.

**Number of patients**	**Method**	**CFR**	**Reference**
17 (HTX)	DI	5.0 ± 0.3*	[11]
26 (HTX)	DI	5.2 ± 1.3*	[12]
18 (young subjects)	PET	4.1 ± 0.9	[13]
22 (elderly subjects)	PET	3.0 ± 0.7	[13]
10	D.TTE	4.5 ± 0.9	[14]
10	PET	4.1 ± 1.0	[15]
10	D.TTE	5.2 ± 1.6	[16]
19	D.TTE	3.7 ± 0.7	[17]
26 (athletes)	D.TTE	5.9 ± 1.0	[17]

* intracoronary papaverine

CFR = coronary flow reserve; DI. = invasive intracoronary Doppler, D.TTE = noninvasive transthoracic Doppler echocardiography, HTX = routine assessment of coronary flow reserve early after heart transplantation

Before defining the normal value of CFR, we should identify appropriately reference groups of healthy subjects. In order to achieve this aim, non invasive studies present a particularly useful approach because we may recruit any subject who gives informed consent to examination. The perfect candidate is a healthy volunteer without cardiovascular signs/symptoms and/or risk factors for both vascular dysfunction and coronary artery disease. In contrast, invasive studies are performed in subjects who complain about chest pain or other cardiac symptoms, implying that coronary microvascular vasodilator dysfunction may limit coronary blood flow and determines angina despite normal coronary epicardial arteries. Two studies [18,19] have shown that such a coincidence is quite frequent. Bearing in mind this limitation it has been proposed by Baumgart et al [20] that in invasive measurements normal limits of CFR may be derived only from highly selected subjects according to the following restricted criteria:

- truly normal epicardial arteries confirmed by intracoronary ultrasound examination

- age <50 years

- absence of symptoms (in addition, we propose an obligatory absence of risk factors for vascular/endothelial dysfunction).

These highly-selected subjects (table 2) exhibit CFR markedly exceeding the cut-off point of 3.0, this value is near to the highest value obtained in an athletes' populations (CFR > 5.0).

The recruitment of reference controls subjects with normal epicardial coronary arteries verified by intracoronary ultrasound is crucial as indicated by the following example. Positive exercise myocardial scintigraphy, primarily considered as false positive in relation to angiographically normal coronary vessels, may frequently turn out to be true positive when control intracoronary ultrasound reveals vascular lesions [21]. In this clinical setting, CFR is a fairly good predictor of "soft lesions", non-visualizable even by coronary angiography [21].

Baumgart [20] proposed a range of age <50 years since PET revealed a significantly higher CFR in younger than in the elderly subjects (Table 2). Other investigators [13] demonstrated that aging-induced reduction of CFR is a result of increased coronary blood flow at rest, whereas maximal blood flow remained relatively unchanged during the years. Only over 70 years, maximal coronary blood flow is reduced. According to these findings [20], the lower limit of normal CFR should be reasonably fixed to 3.0 for subjects up to the age of 50.

## Factors limiting coronary flow reserve

**Figure 3:**
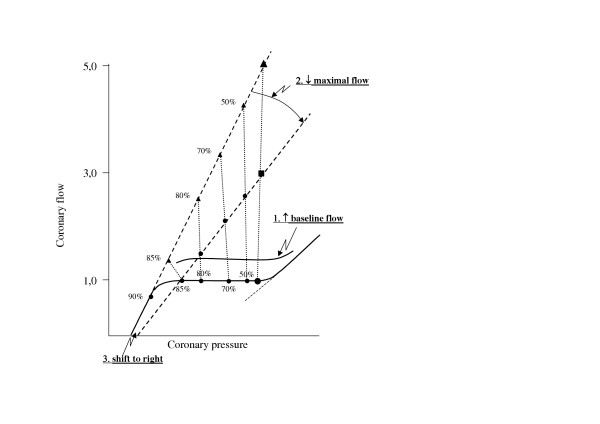
Complexity of CFR concept. Percent values on the curves represent the severity of coronary epicardial stenosis.

In general, the reduction of CFR may be associated with three main kinds of abnormalities [2,3,22,23] (Table 3, Figure 3) and it is even possible for two factors to co-operate simultaneously. However, it has to be taken into account that epicardial coronary artery stenosis, the most visible factor of patients with angina pectoris (examined alone by coronary angiography in daily practice), is only one possible determinant in contrast to several other multi-factorial mechanisms involving coronary microvascular dysfunction. Structural changes (remodelling) in coronary microcirculation can themselves be responsible of CFR reduction. By using myocardial biopsy, some studies have provided the opportunity to compare pathomorphological changes of coronary microcirculation and CFR reduction, documenting the relationship between morphological and hemodynamic abnormalities. In patients with hypertrophic cardiomyopathy, a reduced arteriolar lumen was associated to a reduced CFR [24,25]. Also in hypertensive patients [26] reduced CFR correlated with increased coronary arteriolar wall thickness, i.e, arteriolar remodelling. A decreased arteriolar wall/lumen ratio correlates with reduction of CFR as well as with abnormalities of derived parameters as coronary resistance reserve. All together, these studies strongly support the hypothesis that the microvascular factor is a further important contributor (as extravascular, myocardial factor, i.e. LV hypertrophy, excessive intramyocardial pressure) of CFR reduction. From a pathophysiological point of view, coronary microvascular disease is associated with alternative ischemic cascade where stress test induces chest pain, ECG ST-segment depression and myocardial scintigraphic perfusion defect despite the lack of changes in echocardiographic-derived regional myocardial wall motion [27].

**Table 3:** Three groups of factors limiting CFR:

**1. Increase of resting coronary blood flow due to increased myocardial oxygen demand as a result of:**
• tachycardia
• increased myocardial contractility
• myocardial hypertrophy
**2. Decrease of maximal (hyperemic) coronary blood flow:**
• epicardial coronary artery stenosis
• decrease mean aortic pressure = coronary perfusion pressure e.g. aortic insufficiency, exaggerate response to vasodilator agent
• wall thickening (remodeling) of resistance arterioles
• reduced density of arterioles
• cardiomyocyte hypertrophy
• perivascular fibrosis
• interstitial fibrosis
• endothelial dysfunction
• increased blood viscosity: policythemia, macroglobulinemia
• elevated LV diastolic pressure increasing extravascular compressive forces and resistance (particularly in subendocardial layer).
**3. Shift to the right in the pressure-flow relation through maximally dilated vessels due to an increase in zero flow pressure line:**
• increased left ventricular diastolic pressure
• tachycardia
• myocardial hypertrophy

## How much coronary microvascular disease may reduce CFR?

In a recent study Voci et al. [28] minimised the role of reduced microvascular CFR, probably underestimating the unfavourable influence of impaired coronary microcirculation on prognosis. These authors stated that patients die of epicardial coronary artery disease, not of microvascular disease. In other studies [29-35], however, patients with no or minimal coronary stenosis of epicardial coronary arteries exhibited a significantly blunted CFR in relation to microvascular abnormalities induced by various cardiovascular diseases (Table 4). Several patients had markedly reduced CFR < 2.0 and in some pediatric patients with hypertrophic cardiomyopathy CFR was <1.0 [29]. On these grounds, the authors [29] postulated that non-hypertrophic free wall steals the blood flow from the hypertrophied septum after a vasodilator infusion. A gradual decrease of CFR, parallel to more advanced stages of microvascular disease, was observed in diabetic patients, patients with syndrome X and also hypertensive patients without overt coronary artery stenosis (Table 4). In this view it is notable the experience of Rigo [36], who collected CFR values measured by transthoracic Doppler echocardiography in large population samples affected by various cardiac diseases (Table 5). It is also of interest that the reduction of CFR is reversible in some cases, for instance after regression of LV hypertrophy subsequent to aortic valve replacement in patients with aortic valve stenosis, after anti-hypertensive treatment or even after cholesterol treatment [37-42]. (Table 6). Importantly, even drugs of the same group (angiotensin converting enzyme inhibitors [perindopril versus enalapril] and statins [simvastatin versus pravastatin]) exhibited different influence on CFR (Table 6). After simvastatin treatment [39], the percent increase of CFR correlated with percent decrease of cholesterol and triglycerides levels.

**Table 4:** CFR in microvascular disease with normal coronary angiogram.

**Clinical setting**	**CFR**
HCM pediatric pts (septum) [29]	0.84 ± 0.33
Control	2.94 ± 0.35
Aortic insufficiency [30]	1,67 ± 0,4
Control	4,03 ± 0,52
Dilated cardiomyopathy [31]	2.2 ± 0.8
Control	3.3 ± 0.8
Dilated cardiomyopathy [32]	2,0 ± 0,6
NYHA class I	2,43 ± 0,4
NYHA class II	2,21 ± 0,2
NYHA class III	1,98 ± 0,3
NYHA class IV	1,78 ± 0,3
Control	3,2 ± 0,5
Diabetes [33]	
Without retinopathy	2.8 ± 0.3
with early diabetic retinopathy	2.3 ± 0.3
with slightly advanced retinopathy	1.6 ± 0,2
Control	3.3 ± 0,4
Patients with chest pain and [34]	
Without ST depression in ECG exercise test	3.0 ± 0.6
With up-slope ST depression in ECG exercise test	3.1 ± 0.6
With flat ST depression in ECG exercise test	2.1 ± 0.6
With down-slope ST depression ECG exercise test	2.0 ± 0,4
Hypertension [35]	
Concentric remodeling	2.0 ± 0.7
Concentric hypertrophy	2.3 ± 0.8
Eccentric hypertrophy	2.9 ± 0.6
Normal geometry	2.7 ± 0.4
Control	4.2 ± 0.5

**Table 5:** Findings of CFR in some diseases associated to coronary microvascular dysfunction and in healthy controls [36]

**Clinical setting**	**CFR**
Hypertrophic cardiomyopathy	2.21 ± 0.2
Dilated cardiomyopathy	1.9 ± 0.2
Syndrome X	2.27 ± 0,3
Control group	3.3 ± 0.3

**Table 6:** Increase in CFR in microvascular disease after treatment.

**Disease**	**CFR**	**Effect of treatment**	**p**
Aortic stenosis [37]	1.8 ± 0.5	2.6 ± 0.7 (valve replacement)	<0.05
Familial hypercholesterolemia [38]	2.3 ± 0.6	3.3 ± 1.2 (simvastatin)	<0.05
Hypercholesterolemia [39]	2.4 ± 0.7	3.2 +1.2 (simvastatin)	<0.05
	2.2 ± 0.7	2.3 ± 0.6 (pravastatin)	>0.05
Arterial hypertension [40]	1.9 ± 0.31	2.1 ± 0.3 (nebivolol)	<0.05
Arterial hypertension [41]	2.1 ± 0.6	3.5 ± 1.9 (perindopril)	<0.05
Arterial hypertension [42]	2.4 ± 0.7	2.4 ± 0.6 (enalapril)	>0.05
	2.7 ± 0.8	3.7 ± 1.8 (verapamil)	<0.05

Interestingly, in patients with epicardial coronary artery stenosis and severe hypercholesterolemia, single LDL-apheresis improved microcirculation by increasing CFR from 1.91 ± 0,68 to 2.48 ± 0.68 [43]. This finding demonstrates that, even after single LDL-lowering intervention, some patients can move quickly from a group where PTCA appears to be required (corresponding to CFR < 2.0) to a group with CFR >2.0, where PTCA may not be needed anymore.

## Vasodilators inducing hyperemia

Two main pharmacological vasodilators, adenosine and dipyridamole, are used in humans to recruit CFR. The characteristics of these agents are compared in Table 7. These agents have an advantage over exercise and dobutamine, which represent submaximal stimuli for coronary flow reserve and are much more technically demanding for ultrasound imaging of CFR [44]. Either adenosine or dipyridamole were used in referred studies of Tables 4, 5, where only studies with control group are reported. The control group provides an opportunity to compare how much CFR is reduced (sometimes more than 50%) in patients with different involvement of coronary microvascular disease.

**Table 7:** Comparison between adenosine and dipyridamle characteristics

	Adenosine	Dipyridamole
Duration of action	30 sec	30 min
Time to max. Effect	30–55 sec	6–16 min
Advantage	Short action, short-lasting adverse effects	prolonged action allow to assess CFR and wall motion abnormalities during the same examination
Disadvantage	Frequent- hyperventilation Rare – bradycardia, AV block, hypotension, flushing, headache,	possibility of antidote-resistance prolonged ischemia, hypotension, flushing, headache, hyperventilation,

## Prognostic value of impaired microvascular CFR

The prognostic role of impaired microvascular CFR has been found unfavourable. In a study by Marks at al. [45], reduced CFR due to unspecified microvascular disease predicted increased mortality – 20% vs. 7% in a group with normal CFR (p < 0.016). The relationship between unfavourable prognosis and reduced CFR in patients with hypertrophic or idiopathic dilated cardiomyopathies was recently reported [46,47]. In other two studies where patients were divided according to CFR tercentyle [48] or maximally stimulated coronary blood flow tercentyle [49], the subgroup defined as the lowest tercentyle had the worst outcome. The markedly blunted maximal blood flow was related with poor prognosis not only in HCM patients [49] but also in patients with idiopathic dilated cardiomyopathy (DCM) [50]. In this clinical setting, strongly depressed dipyridamole-stimulated maximal coronary blood flow was associated, with a 3.5 relative risk of death or development or progression of heart failure. These results support the hypothesis that chronic myocardial hypoperfusion or repetitive myocardial ischemia attributable to abnormal coronary microcirculatory flow could exert a detrimental role in the evolution of idiopathic LV dysfunction toward overt DCM. Cecchi et al. [49] hypothesised that coronary microvascular dysfunction may represent a common pathway leading to a disease progression in different cardiomyopathies, including conditions as aortic valve stenosis and hypertensive heart disease.

## Limitations and hypothesis

In several of the referred reports, dipyridamole was used to produce vasodilating hyperemia. Adenosine (140 ug/kg/min) has been shown to be either similar [51] or more potent vasodilator agent [52] than high-dose dipyridamole (0.84 mg/Kg). In relation to the possibility that dipyridamole-recruited CFR could be submaximal, we can not be absolutely sure about the appropriateness of the lower limit of normal CFR = 3.0. To resolve this problem, apart from vasodilator selection, the choice of appropriate control groups is also due, by excluding smokers and patients with arterial hypertension, hyperlipidemia, obesity, diabetes mellitus, and, possibly, those affected by hyperhomocysteinemia. These highly selecting criteria probably was not fulfilled in previous studies, in particular the oldest, where the newest recognized factors limiting CFR were not yet known. Recently, new evidence has been given that in healthy subjects even single exposition to passive smoking [53], fat meal inducing hypertriglyceridemia [54], hyperhomocysteinemia [55], and estrogen decrease in menstrual phase of cycle [56] can reduce CFR by approximately 30%. Thus, we can not be certain that normal CFR starts from 3.0 because CFR values higher than 5.0 were recorded in humans. Interestingly, in a transplanted heart which can not be equivalent of intact heart in healthy volunteers, CFR may also achieved more 5.0 (see Table 2). This high value is probably the result of intracoronary papaverine, which is not used currently. However, these findings strongly supports the hypothesis that CFR may achieve much higher level than 3.0.

As regard validation of a non-invasive methods, transthoracic Doppler echocardiography closely agrees with intracoronary Doppler flow wire results in assessing CFR. Good correlation of non-invasive and invasive Doppler assessment of CFR has been shown both in LAD [57] and RCA [58]. The feasibility of transthoracic Doppler echocardiography to detect coronary flow is 80–98% in LAD, 50–87% in RCA and 43–72% in Cx [6,36,59].

## Conclusion

Isolated coronary microvascular abnormalities are overt by reduced CFR despite normal epicardial coronary arteries. These abnormalities may occur in several diseases (arterial hypertension, diabetes mellitus, hypercholesterolemia, syndrome X, aortic valve disease, hypertrophic cardiomyopathy and idiopathic dilated cardiomyopathy). The prognostic role of impaired microvascular CFR has been shown unfavourable, in particular in patients with hypertrophic or idiopathic dilated cardiomyopathies. CFR reduction may be reversible, for instance after regression of left ventricular hypertrophy subsequent to valve replacement in patients with aortic stenosis, after anti-hypertensive treatment or using cholesterol lowering drugs. CFR may increase by 30% or more after pharmacological therapy and achieve level >3.0. In contrast to other non invasive tools as PET, very expensive and associated with radiation exposure, transthoracic Doppler-derived CFR is equally non invasive but cheaper, very accessible [60] and prone to a reliable exploration of coronary microvascular territories, not detectable by invasive coronary angiography, able to visualize only large epicardial arteries.
